# *In vitro* evaluation of total mixed ration supplemented with exogenous fibrolytic enzymes for crossbred cows

**DOI:** 10.14202/vetworld.2017.281-285

**Published:** 2017-03-05

**Authors:** Pravin Mohan Lunagariya, Ram Sharan Gupta, Subhash Parnerkar

**Affiliations:** 1 Livestock Research Station, College of Veterinary Science and Animal Husbandry, Anand Agricultural University, Anand, Gujarat, India; 2 Department of Animal Nutrition Research, College of veterinary Science and Animal Husbandry, Anand Agricultural University, Anand, Gujarat, India

**Keywords:** exogenous fibrolytic enzymes, *in vitro* digestibility, metabolizable energy, microbial biomass production, total gas production

## Abstract

**Aim:**

The study was conducted to evaluate the levels of exogenous fibrolytic enzymes (EFE) on *in vitro* digestibilities of dry matter (DM) and organic matter (OM), total gas production (TGP), metabolizable energy (ME) content, and microbial biomass production (MBP).

**Materials and Methods:**

The total mixed ration (TMR) was prepared using 30% each of sorghum hay and groundnut straw and 40% compound concentrate mixture to meet nutritional requirement of cow (500 kg) producing 12 kg fat corrected milk. The EFE was incorporated at 0, 40, 60, 80, 100, 120, 140, 160, 180, 200, 220, 240, 260, 280, 300, 320, 340, 360, 380, and 400 mg/kg TMR. The TMR substrates with different levels of EFE were *in vitro* incubated to ascertain their effect on digestibility, gas production, and nutritive values.

**Results:**

The significantly (p<0.05) higher and optimum *in vitro* digestibilities of DM (63.03%) and OM (63.62%) as well as TGP (72.35 ml/500 mg TMR) were observed at supplementation of 240 mg EFE/kg TMR, while ME (7.16 MJ/kg DM) and MBP (97.63 mg/500 mg TMR) were also better.

**Conclusion:**

The incorporation of EFE at 240 mg/kg TMR resulted significantly (p<0.05) higher and optimum *in vitro* digestibilities of DM and OM. The TGP, ME, and MBP were also better. The levels of EFE 240 mg/kg TMR were found suitable for further *in vivo* study in crossbred cows.

## Introduction

In India, ruminant feeding relies mainly on agro-industrial byproduct and crop residues. These feeds are low in energy and protein owing to high fiber, lignin and silica. Ruminant animals have the ability to convert low quality feeds into high quality protein due to ruminal microorganisms that synthesize and secrete β 1-4 cellulase enzyme complex, thereby allowing hydrolysis of plant cell wall components. However, the actual conversion of feeds, especially fibrous forages to meat and milk, is not very efficient. Only 10-35% of energy intake is captured as net energy because 20-70% of cellulose may not be digested by the animal [1].

The supplementation of exogenous fibrolytic enzymes (EFE) in feeds has been identified as a promising biological treatment to improve the energy availability of feeds to ruminants. Positive effects of EFE include direct hydrolysis, improvement in palatability, changes in gut viscosity, complimentary action with ruminal enzymes, change in the site of digestion [2], increase in rumen bacterial colonization of the substrate [3], altering fiber cell wall structures, and thinning of fiber cell wall [4]. The feeding of diets supplemented with EFE were shown to improve the digestibilities of dry matter (DM), organic matter (OM), crude fiber, neutral detergent fiber (NDF), cellulose and hemicellulose in dairy animals [5-7]. An *in vivo* investigation need living animals which is expensive as well as time consuming. An *in vitro* study conducted outside living animals to evaluate the effect of EFE in improving ration digestibility as they are less expensive, less time consuming and allows more control [8].

It was hypothesized that EFE improve the fermentation kinetics. The study was planned to assess the effect of supplementing various levels of EFE in TMR on *in vitro* digestibility, total gas production (TGP), metabolizable energy (ME) content, and microbial biomass production (MBP).

## Materials and Methods

### Ethical approval

An ethical approval was obtained from the Institutional Animal Ethics Committee of College of Veterinary Science and Animal Husbandry, Anand Agricultural University, Anand for this study. The study was conducted at Animal Nutrition Research Department, College of Veterinary Science and A. H., Anand Agricultural University, Anand.

### Sample preparation, *in vitro* incubation and analysis

Sorghum hay, groundnut straw and compound concentrate mixture were procured from Animal Nutrition Research Department, College of Veterinary Science and Animal Husbandry, Anand Agricultural University, Anand. These ingredients were oven dried at 70°C and finely ground in mill using 1 mm sieve. These ingredients were mixed in ratios of 30%, 30% and 40% to prepare total mixed ration (TMR). The TMR was prepared to meet nutritional requirement of dairy cow (500 kg) producing 12 kg 4% fat corrected milk (FCM) per day [9]. The calculated nutritional values of TMR were 10.61% crude protein (CP), 6.59% digestible CP (DCP), 56.51% total digestible nutrients, and 2.05 Mcal ME/kg DM. The ingredients and TMR were analyzed for proximate constituents [10], fiber fractions [11] and calcium and phosphorus.

The EFE was procured from M/s DSM Nutritional Products India Pvt. Ltd., Thane, India. It contained endo 1,4-β glucanase 800, 1(3),4-β glucanase 700 and endo 1,4-β xylanase 2700 IU/g. The EFE was incorporated at 0, 40, 60, 80, 100, 120, 140, 160, 180, 200, 220, 240, 260, 280, 300, 320, 340, 360, 380, and 400 mg/kg TMR to know their effect on *in vitro* digestibility of DM and OM, *in vitro* TGP, ME content and MBP. The level of EFE used was designated as E_0_, E_4_, E_6_, E_8_, E_10_, E_12_, E_14_, E_16_, E_18_, E_20_, E_22_, E_24_, E_26_, E_28_, E_30_, E_32_, E_34_, E_36_, E_38_ and E_40_, respectively.

Rumen liquor was collected from three crossbred cows using stomach tube. The cows were fed individually to meet nutrients requirement [9] with free water access. The rumen liquor was strained through four layer muslin cloth and was termed strained rumen liquor (SRL). TMR with various levels of EFE were incubated for 48 h in quadruplet at 39±1°C for 48 h in a shaker twin water bath with 40 ml of fresh McDougall buffer and 10 ml SRL as per Menke *et al*. [12]. After incubation, the content of each syringe was filtered through dried and pre-weighed Gooch crucible, which was again dried and weighed. Simultaneously, the blank was also run without TMR sample in quadruplet. The *in vitro* TGP was taken after subtracting gas production from blank. The ME [13] and MBP [14] were calculated as:

ME (MJ/kg DM)= 2.20+0.136 Gp+0.057 CP%, (R^2^=0.94)

Where, CP is crude protein% and Gp is ml of net gas production from 200 mg dry sample.

MBP= {TDOM-(2.2×net gas volume)}; TDOM=(Feed OM incubated-residue OM).

Where, TDOM is total digestible OM.

### Statistical analysis

The data on digestibility, TGP, ME and MBP were analyzed [15] using Duncan’s multiple range tests (SPSS 9.00 software).

## Results and Discussion

The data for proximate composition and fiber fractions of ingredients and TMR used are presented in Table-1. The TMR contained 10.24% CP, 55.06% NDF, and 28.82% ADF. The data for *in vitro* digestibility (%) of DM and OM, TGP, ME content and MBP of TMR incorporated with various levels of EFE are presented in Table-2.

**Table-1:** Average proximate composition and fiber fractions (% on DM basis) of feeds and fodder.

Parameters	TMR	Concentrate mixture	Sorghum hay	Groundnut straw
CP	10.24±0.22	14.18	5.72	9.02
EE	3.36±0.05	6.27	1.72	1.05
CF	21.79±0.40	9.96	35.18	28.98
NFE	53.52±0.24	59.79	47.92	50.05
Total ash	11.09±0.10	9.80	9.46	10.90
Silica	3.10±0.44	1.08	3.24	2.07
Calcium	1.24±0.36	1.31	0.70	0.91
Phosphorus	0.48±0.01	0.65	0.27	0.36
NDF	55.06±0.87	26.48	72.52	69.91
ADF	28.82±0.27	11.19	48.16	36.61

DM=Dry matter, TMR=Total mixed ration, CP=Crude protein, NDF=Neutral detergent fiber, ADF=Acid detergent fiber, NFE=Nitrogen-free extract, CF=Crude fiber, EE=Ether extract

**Table-2:** Average IVDMD, IVOMD, IVTGP, ME and MBP of TMR containing different levels of exogenous fibrolytic enzymes.

TMR	Particulars				

	IVDMD (%)	IVOMD (%)	IVTGP (ml)	ME (MJ/kg DM)	MBP (mg/500 mg TMR)
E_0_	54.36^a^±0.45	55.44^a^±0.36	61.30^a^±1.05	6.49^a^±0.06	87.37^a^±1.37
E_4_	54.65^a^±0.62	55.63^a^±0.58	61.40^a^±1.17	6.49^a^±0.07	87.18^a^±0.72
E_6_	54.82^ab^±0.78	56.26^ab^±0.59	62.00^a^±1.23	6.53^ab^±0.07	88.89^a^±0.75
E_8_	55.36^ab^±0.25	55.94^a^±0.27	62.73^ab^±1.45	6.58^ab^±0.09	87.16^a^±1.79
E_10_	55.54^ab^±0.45	56.86^ab^±0.43	64.03^abc^±0.84	6.65^bc^±0.05	87.26^a^±0.56
E_12_	56.54^b^±0.58	57.76^bc^±0.49	65.60^bcd^±0.56	6.75^cd^±0.03	87.56^a^±0.82
E_14_	56.67^b^±0.64	58.65^c^±0.58	65.88^bcd^±0.46	6.77^cd^±0.03	89.72^ab^±1.50
E_16_	58.43^c^±0.20	59.18^c^±0.12	67.18^cde^±0.64	6.84^de^±0.04	89.08^a^±1.50
E_18_	59.46^cd^±0.31	60.96^d^±0.11	68.45^def^±0.45	6.92^ef^±0.03	93.27^bc^±0.86
E_20_	60.36^de^±0.40	62.28^de^±0.29	70.08^efg^±0.29	7.02^fg^±0.02	95.96^cd^±0.39
E_22_	62.05^e^±0.36	63.62^ef^±0.44	70.85^fg^±0.61	7.07^g^±0.02	100.71^f^±1.36
E_24_	63.03^ef^±0.39	63.96^f^±0.24	72.35^g^±0.56	7.16^g^±0.03	97.63^def^±1.00
E_26_	63.21^f^±0.39	63.90^ef^±0.13	72.33^g^±0.66	7.16^g^±0.04	96.29^cde^±1.22
E_28_	63.12^f^±0.22	64.68^f^±0.07	72.28^g^±0.28	7.15^g^±0.02	100.42^ef^±0.82
E_30_	63.74^f^±0.57	64.44^f^±0.63	72.78^g^±0.82	7.18^g^±0.05	99.12^def^±1.24
E_32_	63.55^f^±0.50	64.28^f^±0.67	72.20^g^±0.67	7.15^g^±0.04	98.60^def^±1.98
E_34_	63.56^f^±0.46	64.54^f^±0.48	72.50^g^±0.67	7.17^g^±0.04	99.49^def^±1.09
E_36_	63.59^f^±0.31	64.78^f^±0.51	72.35^g^±0.81	7.16^g^±0.05	101.14^f^±2.47
E_38_	63.20^f^±0.42	64.61^f^±0.20	72.00^g^±0.50	7.14^g^±0.03	100.74^f^±1.19
E_40_	63.75^f^±0.16	64.89^f^±0.20	72.25^g^±0.87	7.15^g^±0.03	100.53^f^±1.40
SEM	0.41	0.41	0.50	0.03	0.67

a, b, c, d, e, f, g Means with different superscripts in a column for a parameter differ significantly (p<0.05). DM=Dry matter, TMR=Total mixed ration, MBP=Microbial biomass production, ME=Metabolizable energy, IVTGP=*In vitro* total gas production, IVOMD=*In vitro* organic matter digestibility, IVDMD=In vitro dry matter digestibility

### *In vitro* DM digestibility (IVDMD)

The data revealed significant (p<0.05) effect of level of incorporation of EFE at and beyond 120 mg/kg TMR (56.54±0.58%) in comparison to control TMR (54.36±0.45%) without EFE. However, incorporation level of EFE at 240 mg/kg TMR had improved (p<0.05) DM digestibility (60.36±0.39%) than lower levels of incorporation. Further increase in level of fibrolytic enzyme did not show beneficial effect on IVDMD.

Incorporation of EFE at 2.5 g/kg DM TMR (roughage to concentrate ratio of 60:40) resulted in similar observations and significantly higher 62.18% (p≤0.001) IVDMD than control TMR (58.44%) [16]. Miachieo and Thakur [5] reported optimum and higher IVDMD of TMR having concentrate, wheat straw and green oats in ratios of 40:40:20 as 55.0% when incorporated with fibrolytic enzymes at 1.5 g/kg DM than control TMR without EFE or TMR with higher (3.0 g/kg DM) EFE. These findings were in accordance with this study. Similarly, significantly higher IVDMD was also observed [17-19] when TMR was supplemented with fibrolytic enzyme. However, the effect of incubation of corn silage based diet with exogenous enzyme at 1.68 and 2.52 g/kg was nonsignificant on apparent DM degradability [20] as silage is considered as pre-digested forage (corn silage).

### *In vitro* OM digestibility (IVOMD)

The data of IVOMD revealed significant (p<0.05) effect of level of incorporation of EFE at and beyond 120 mg/kg TMR (57.76±0.49%) in comparison to control (55.44±0.36%), but optimum and significantly higher values were observed (63.96±0.24%) when EFE incorporated at 240 mg/kg TMR and further increase in level of EFE had no beneficial effect.

The quadratic improvement (p=0.004) of *in vitro* OM degradability was observed as 0.336, 0.365, 0.387 and 0.426 g/g DM, respectively, when sorghum straw was incubated with 0, 6, 12 and 24 mg enzyme/g DM [21]. The optimum IVOMD (p<0.05) reported was 56.1% for TMR incorporated with EFE at 1.5 g/kg DM than for control or higher levels of EFE (3.0 g/kg DM) in TMR [5]. The findings of these studies were in agreement with present experiment. Similarly, *in-vitro* OM disappearance of alfalfa hay (46.21%) was optimum (p<0.01) when fibrolytic enzyme was added at 2 μl/g DM compared with alfalfa hay (44.9%) incubated without EFE. Intermediate effect was observed for alfalfa hay incubated with 0.5, 1.0 and 1.5 µl EFE/g DM, but OM disappearance was not improved when alfalfa silage and barley silage were used [22] being pre-digested fodder. An improvement in OM degradability was also reported on supplementation of fibrolytic bacterial culture in wheat straw DM [23] and fibrolytic enzymes in TMR [13] than control.

### *In vitro* TGP (IVTGP)

There was a significant difference in gas production for control (61.30±1.05 ml) and experimental TMRs incorporated with EFE at 120 mg/kg TMR (65.60±0.56 ml) and higher levels. However, incorporation of EFE at 240 mg/kg TMR had shown significant (p<0.05) effect on IVTGP, and further incorporation of higher levels of EFE in TMR had not shown further improvement in TGP.

The higher (p≤0.05) *in vitro* gas production observed as 96.33 ml and 96.00 ml when dry sorghum supplemented with EFE (1:1 mixture of neutral cellulase-3000 units/g and fungal xylanase-200000 units/g) at 0.6 and 0.8%, respectively, than dry sorghum supplemented without or with lower levels of fibrolytic enzymes (0, 0.01, 0.1, 0.2, 0.3, and 0.5%). Supplementation of higher levels (0.9% and 1%) of fibrolytic enzymes had no further beneficial effect on *in vitro* gas production [24]. Similarly, significant effect of EFE supplementation in roughage diet [18], maize stover and sugarcane bagasse [25], and sorghum straw [21] was observed for *in vitro* gas production.

An *in vitro* gas production (137.14-230.97 ml/g DM) at 48 hours of incubation had shown no incremental effect when five TMR of different maize silage (F) to concentrate (C) ratio (0F:100C, 25F:75C, 50F:50C, 75F:25C, 100F:0C) supplemented with EFE, *viz*., cellulase 1 µl/g (C, 0.033 unit/g), xylanase 1 µl/g (X, 0.038 unit/g), a mixture of cellulase, and xylanase (XC, 1:1, v:v) was used [13] this may be due to utilization of pre-digested forage (maize silage).

### ME content of feed

The ME content of TMR ranges from 6.49 to 7.18 MJ/kg DM. However, ME of TMR (6.65±0.05 MJ/kg DM) significantly improved at EFE level 100 mg/kg than control but optimum and significantly higher ME content of TMR was 7.16±0.03 MJ/kg DM when TMR was incorporated with EFE 240 mg/kg. Further higher level of incorporation of EFE had shown no improvement in ME content of TMR. The ME content of TMR was lower than calculated ME (9.50 MJ/kg DM), but ME content of TMR increased as there was increase in level of incorporation of EFE.

The ME content of sorghum straw increased (p<0.05) quadratic and values were 5.0, 5.4, 5.7 and 6.3 MJ/kg DM, respectively, when incubated with exogenous enzymes mixture at 0, 6, 12 and 24 mg/g DM [21] levels. The TMR having 50% maize silage (F) and 50% concentrate when incubated with cellulase 1 µl/g (C, 0.033 unit/g), xylanase 1 µl/g (X, 0.038 unit/g), a mixture of cellulase and xylanase (XC, 1:1, v:v) and without EFE resulted in similar and nonsignificant (p=0.0734) ME content of TMR as 6.89, 6.76, 7.12 and 6.88 MJ/kg DM, respectively [13].

### MBP

The MBP (mg/500 mg TMR) increased linearly with increasing levels of EFE in TMR, however, significantly higher MBP was achieved at 180 mg EFE/kg TMR (93.27±0.86). The optimum and significantly higher MBP as 97.63±1.00 and 100.71±1.36 mg/500 mg TMR was achieved at 240 and 220 mg EFE/kg TMR, respectively, than control or lower levels. The further higher levels of incorporation of EFE did not show improvement in MBP.

Higher level of MBP (72.21-90.69 mg/200 mg) was achieved when 0.00-30.00% walnut (*Juglans regia*) cake was incorporated in concentrate to form iso-nitrogenous TMR [14]. The MBP values reported in this study are within the normal range (100-470 g/kg TDOM) for mixed diets [26]. Nonsignificant (p=0.6602) difference in MBP was observed (513.2, 520.3, 496.2 and 521.0 mg/g DM, respectively) when TMR with 50% maize silage (F) and 50% concentrate was incubated with EFE, *viz*., cellulase 1 µl/g (C, 0.033 unit/g), xylanase 1 µl/g (X, 0.038 unit/g), a mixture of cellulase and xylanase (XC, 1:1, v:v), and control TMR [13].

*In vitro* digestibility of DM and OM and IVTGP was found to be optimum (p<0.05), respectively, as 63.03±0.39%, 63.96±0.44% and 70.85±0.61 ml/500 mg of TMR when incorporated with EFE at 240 mg/kg TMR than control TMR, or other levels of EFE. The optimum and higher ME (7.16±0.03 MJ/kg DM) and MBP (97.63±1.00 mg/500 mg TMR) was achieved at EFE 240 mg/kg TMR in comparison to TMR without EFE. Positive and linear increase in TGP was observed with improvement in digestibility of DM and OM. The same correlation of gas production with digestibility of DM and OM under *in vitro* study were also observed [27,14]. An improved digestibility, gas production, ME and MBP may be due to action of EFE to degrade complex fraction to simple molecules making them more available to rumen microbes [28], synergetic effect of EFE and rumen microbe [29], enhance attachment of rumen microbial to feed particles [30], and stimulatory effect on rumen microbiota [31].

## Conclusion

An *in vitro* digestibility of DM (56.54%), OM (57.76%), and TGP (65.60 ml/500 mg TMR) was significantly (p<0.05) higher when EFE was supplemented at 120 mg/kg TMR in comparison to control, whereas ME (6.65 MJ/kg DM) and MBP (93.27 mg/500 mg TMR) were significant at 100 and 180 mg/kg TMR, respectively. The incorporation of EFE at 240 mg/kg in TMR resulted in significantly (p<0.05) higher and optimum *in vitro* digestibilities of DM (63.03%) and OM (63.96%) compared to lower levels. TGP (72.35 ml/500 mg TMR), ME (7.16 MJ/kg DM), and MBP (97.63 mg/500 mg TMR) were also better. The digestibility, gas production and nutritive values of TMR did not show improvement with higher supplementation of EFE. The levels of EFE 240 mg/kg TMR were found suitable for further *in vivo* study in crossbred cows.

## Authors’ Contributions

RSG and SP: Conceptualized, designed and supervised the study. PML: Designed, executed study, carried out laboratory analysis and drafted and revised the manuscript. All authors have read and approved the final manuscript.
